# Effects of Vibro-Stimulation Ankle Bracing on Tactile Sensation and Center of Pressure Dynamics in Individuals with Chronic Ankle Instability: A Randomized Clinical Trial

**DOI:** 10.3390/healthcare14111518

**Published:** 2026-05-29

**Authors:** Hanieh Khaliliyan, Mahmood Bahramizadeh, Amirhossein Zare, Majid Ansari, Farhad Ghaffari, Arash Sharafatvaziri, Hicham Khabbache, Francesco Chirico, Diego Burzomati, Aldo Sitibondo, Amelia Rizzo

**Affiliations:** 1 Department of Orthotics and Prosthetics, School of Rehabilitation Sciences, Isfahan University of Medical Sciences, Isfahan 8174673461, Iran; haniehkhaliliyan@yahoo.com (H.K.); zareamirhossein75@yahoo.com (A.Z.); 2 Neuromusculoskeletal Rehabilitation Research Center, University of Social Welfare and Rehabilitation Sciences, Tehran 1985713871, Iran; 3 Sports Medicine Research Center, Neuroscience Institute, Tehran University of Medical Sciences, Tehran 1461884513, Iran; majid.ansari@gmail.com; 4 Orthopedic & Rehabilitation Research Center, Shiraz University of Medical Sciences, Shiraz 7134814336, Iran; farhadb75@yahoo.com; 5 Center for Orthopedic Trans-Disciplinary Applied Research, Tehran University of Medical Sciences, Tehran 1461884513, Iran; arash.sharafatvaziri@gmail.com; 6 Laboratory of «Morocco: History, Theology and Languages», Department of Psychology, Faculty of Arts and Human Sciences Fès-Saïss, Sidi Mohamed Ben Abdellah University, Fez 30000, Morocco; hicham.khabbache@usmba.ac.ma; 7 Health Service Department, Italian State Police, Ministry of the Interior, Multifunctional Health Center, Via Augusto Anfossi 2, 20135 Milan, Italy; medlavchirico@gmail.com; 8 Medical Legal Centre of Messina, National Institute of Social Welfare (INPS), 93124 Messina, Italy; diego.burzomati@inps.it (D.B.); aldo.sitibondo@gmail.com (A.S.); 9 Infectious Diseases Unit, University Hospital “G. Martino”, 98124 Messina, Italy

**Keywords:** ankle instability, ankle brace, vibration, somatosensory stimulation, postural control

## Abstract

**Background/Objectives:** Chronic ankle instability (CAI) is a common sequela of lateral ankle sprain and is characterized by recurrent episodes of giving way, sensorimotor deficits, impaired postural control, and diminished functional performance. While exercise-based rehabilitation, including neuromuscular training and proprioceptive exercises, remains the gold standard for managing CAI, patients often require additional support during daily activities. Orthotic interventions predominantly address mechanical instability, yet there is a clinical gap in providing integrated solutions that simultaneously offer mechanical support and sensory feedback to enhance postural control. This study aimed to investigate the effects of a semi-rigid ankle brace combined with vibro-stimulation on tactile sensation and center of pressure excursion in individuals with CAI. **Methods:** A randomized clinical trial was designed with two parallel groups and repeated measurements over time. Thirty adults (n = 15 per group) aged 18–35 years, who met the International Ankle Consortium criteria for CAI, were recruited. Participants in the experimental group received a semi-rigid ankle brace integrated with a wearable vibro-stimulation system, whereas those in the comparison group used the ankle brace alone. Outcome measures were collected at baseline, after 10 min, and after 2 and 4 weeks. Primary outcomes included Vibration Detection Rate and phase plane portraits assessed using a 128 Hz tuning fork and a force plate. **Results:** The ankle bracing plus vibration band group demonstrated significantly greater improvement at 4 weeks than the orthosis group in Vibration Detection Rate (F (3,26) = 31.93, *p* < 0.001, η^2^ = 0.78). Also, the largest effect was observed for the anteroposterior phase plane portrait at 4 weeks (MD = −2.10 ± 0.42, 95% CI: −2.96 to −1.23, *p* < 0.001, d = −1.79). **Conclusions:** The findings suggest that combining a semi-rigid ankle bracing with vibro-stimulation provides additional benefits over the use of bracing alone in individuals with CAI.

## 1. Introduction

The ankle joint sustains the highest load per unit area among human joints [1], serving as a focal point during walking and running and bearing body weight while standing [1,2]. This makes it susceptible to ligamentous injuries, with approximately 85% related to lateral ankle sprain (LAS) [3]. LAS typically occurs during cutting maneuvers, landing tasks, or lateral movements that induce excessive rearfoot supination relative to tibial external rotation [4]. It accounts for about 10–30% of all sports-related injuries [5], with one case occurring per 10,000 individuals daily [6]. Key risk factors include a history of previous LAS and impairments in proprioception and balance [7]. Recurrent LAS episodes may lead to long-term complications such as ankle osteoarthritis and ankylosis [8].

When an acute LAS occurs, primary tissue damage develops, affecting musculoskeletal tissues and nerve endings [9]. This leads to acute symptoms such as pain, inflammation, and restricted range of motion [3]. Without appropriate treatment and rehabilitation, a range of sensorimotor impairments may emerge over time. Sensory impairments following an ankle sprain may include impaired proprioception, intermittent pain, and an altered sense of ankle instability, while kinesiophobia is more appropriately considered a psychological consequence associated with persistent symptoms and perceived instability [9]. Conversely, motor impairments primarily involve muscle weakness, altered movement patterns, and reduced physical activity [10]. Rather than operating in isolation, these deficits interact within a defective sensorimotor cycle, where impaired sensory feedback compromises balance and postural control, which in turn reinforces altered motor outputs. Ultimately, these integrated dysfunctions contribute to the perpetuation of chronic ankle instability (CAI), characterized by recurrent episodes of giving way and repeated sprains persisting for more than one year [9]. In CAI, afferent neural pathways linked to the lateral ankle ligaments become disrupted, resulting in deficits in proprioception and joint position sense [11]. These sensory impairments negatively affect neuromuscular control, postural control, and the kinetics and kinematics of weight-bearing activities [12,13]. Mechanoreceptors on the lateral aspect of the ankle joint are crucial for regulating inversion movements by informing the central nervous system (CNS) about the magnitude and direction of ground reaction forces acting on the joint [14].

Individuals with CAI exhibit altered biomechanical patterns, including increased foot inversion and lateral displacement of the center of pressure (CoP) [15]. To enhance talocrural joint stability, ankle bracing categorized into soft and semi-rigid ankle orthoses (AOs) are used [16]. Semi-rigid double-upright (double-bar) designs are preferred as they restrict pathological inversion and lateral displacement of CoP while preserving sagittal plane dorsiflexion and plantar flexion motion during locomotion [17,18]. However, most previous studies on orthoses have focused on motor outputs, largely overlooking sensory impairments and their impact on movement and postural regulation [16].

Vibration is a mechanism for sensory stimulation, widely used to modulate sensory input and motor control in individuals with postural impairments from sensorimotor deficits [19]. Research suggests that vibration positively affects proprioception by activating afferent neural pathways and generating low-threshold sensory input to CNS [19,20]. Previous studies have examined the effects of localized vibration on static and dynamic postural control [20], ankle joint loading patterns [21], and CoP excursion [20]. There is consensus that vibration, applied as feedback or vibrotactile stimulation, can improve these parameters in individuals with CAI.

While both AOs and vibro-stimulation have shown beneficial effects on functional outcomes in CAI individuals, each intervention has primarily targeted one component of the sensorimotor system: mechanical stabilization (orthoses) or sensory input modulation (vibration). Limited evidence exists regarding the combined application of biomechanical correction and sensory enhancement within an intervention protocol, and the existing studies are limited to passive modifications of orthoses using textured materials [22,23]. This study aimed to investigate the effects of using an AO to correct biomechanical instability along with vibro-stimulation to enhance proprioceptive input on tactile sensation and CoP dynamics in individuals with CAI.

## 2. Materials and Methods

### 2.1. Trial Design, Setting, and Registration

This study was a randomized clinical trial with two parallel groups, using a repeated-measured design to examine within- and between-group interactions over time. The study protocol was approved by the Ethics Committee of the University of Social Welfare and Rehabilitation Sciences, Tehran, Iran (ID: IR.USWR.REC.1403.248). The trial was prospectively registered in the Iranian Registry of Clinical Trials (IRCT) before participant recruitment (registration code: IRCT20240514061793N2). Data collection occurred between 5 March 2025, and 28 February 2026, at the Orthotics and Prosthetics Clinic of the University of Social Welfare and Rehabilitation Sciences. Participants were recruited through public announcements and advertisements distributed in physical medicine and rehabilitation clinics and orthopedic clinics in Tehran Province, Iran.

### 2.2. Enrollment, Allocation, Blinding, and Procedure

Block randomization was used to assign participants randomly to one of the two groups. Blocks were generated using https://www.randomizer.org/ (accessed on 28 March 2026) with a 1:1 allocation ratio and a block size of 4 to minimize predictability [24]. Participants were not blinded to the intervention assignment due to the nature of the interventions. To minimize bias, outcome assessors as well as the statistician were blinded to group allocations throughout the study. The random allocation sequence was generated by an independent researcher who was not involved in recruitment, intervention delivery, or outcome assessment. Allocation concealment was maintained using sequentially numbered, opaque, sealed envelopes prepared in advance. After baseline assessments were completed, the envelopes were opened to assign participants to either the experimental or control group. After the pre- and post-assessments, participants were instructed to wear their orthosis for 6–8 h daily during weight-bearing activities. In the trial group, participants were also instructed to use local ankle vibration therapy every other day for 10 min using the vibration band. A total of six vibration therapy sessions were conducted for each participant. Participants were instructed to maintain their usual daily activities and routine physical exercise regimes throughout the four-week study period. No restrictions were placed on sports participation or recreational activities, provided that the assigned intervention device was worn during all weight-bearing tasks as prescribed. This protocol was designed to evaluate the efficacy of the interventions under conditions reflecting typical functional demand and real-world usage. All assessments were conducted by a single researcher. The pre-intervention assessment was conducted without any intervention. The immediate post-intervention assessment was performed after 10 min of wearing the orthoses in order to evaluate the immediate effects of the intervention while the device was being worn. The follow-up assessments at two weeks and four weeks were conducted without the intervention to investigate the therapeutic effects of the intervention over time.

To monitor adherence to the prescribed intervention protocol, a multi-stage compliance strategy was utilized. Participants were provided with daily logbooks to record the duration of orthosis wear and vibration system usage. Furthermore, research staff conducted weekly follow-up phone calls with each participant. Additionally, to verify the correct application and usage of the vibro-stimulation system, participants were requested to send short video recordings of themselves using the device at random intervals every two weeks. Regarding the vibration sessions (10 min every other day), participants recorded these in their logs as binary ‘completed’ or ‘not completed’ tasks. While we monitored the general consistency of orthosis wearing time through weekly interviews to ensure there were no significant behavioral differences between the groups, participants did not record their wearing time on a minute-by-minute basis.

### 2.3. Participants

A total of 30 individuals with CAI (12 males and 18 females; mean age: 20.45 ± 1.8) were recruited for this trial. Following referral, participants were screened for eligibility based on the study’s criteria and allocated to one of two groups. All participants were aged 18 to 35 years. Inclusion criteria aligned with the diagnostic standards for CAI defined by the International Ankle Consortium [25]. Participants needed to have experienced at least one significant LAS within the 12 months prior to enrollment, accompanied by clinical signs such as pain, swelling, and reduced range of motion lasting at least one day. Additionally, they had to report at least two episodes of perceived ankle instability or recurrent sprains within the six months preceding enrollment and no ankle sprain within the 3 months immediately preceding the study. Giving-way was defined as a sudden, uncontrollable episodes of the ankle joint shifting, necessitating a reduction in physical activity, while recurrent sprain was defined as an injury requiring clinical evaluation or resulting in at least one day of missed physical activity. The presence of ankle instability was assessed using the Cumberland Ankle Instability Tool (CAIT) and/or the Foot and Ankle Ability Measure (FAAM). The CAIT consists of 9 items with a total score ranging from 0 to 30, where lower scores indicate greater instability. The FAAM includes two subscales: the Activities of Daily Living (ADL) subscale, with scores ranging from 0 to 64, and the Sports subscale, with scores ranging from 0 to 32, with higher scores reflecting better functional status. A CAIT score of less than 24, an FAAM-ADL subscale score of less than 90%, and an FAAM Sports subscale score of less than 80% indicated CAI. Mechanical ankle stability was evaluated using the anterior drawer test and the talar tilt test. The Persian-adapted versions of CAIT and FAAM were used in this study. Previous psychometric evaluations have demonstrated excellent reliability and validity for both instruments. The CAIT has shown an intra-class correlation coefficient (ICC) of approximately 0.93 with a 95% confidence interval ranging from 0.87 to 0.96 [26], while the FAAM has demonstrated an ICC of about 0.91 with a 95% confidence interval between 0.85 and 0.94 [27].

Exclusion criteria included underlying conditions that could impair balance, including: (1) a history of lower limb surgery; (2) previous fractures of the lower extremity; (3) limb length discrepancy; (4) use of rehabilitation interventions within six months prior to participation; and (5) the presence of spinal or lower-extremity deformities [17]. Participants were also withdrawn if they sustained an acute injury during the study period that affected balance or if any neuromusculoskeletal disorder was diagnosed by a physician.

### 2.4. Interventions

**Figure 1:**
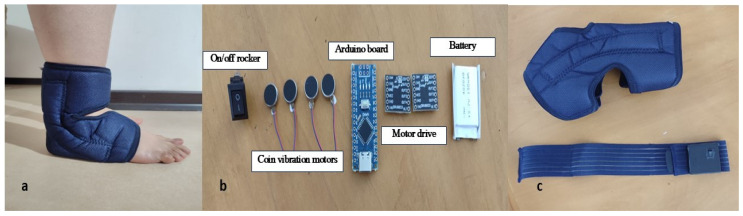
Components of the vibro-stimulation ankle orthosis: (**a**) ankle orthosis; (**b**) vibration system components; (**c**) ankle orthosis with vibration strap.

The intervention protocol for the experimental group involved participants wearing the semi-rigid ankle orthosis equipped with an active vibro-stimulation unit. In contrast, the control group participants wore the same model of semi-rigid ankle orthosis; however, it lacked the vibration mechanism. The trial group intervention had two active and passive parts, but the comparison group intervention had only the passive part. The active part was a vibration system, and the passive part was an AO. The AO was designed to provide semi-rigid mechanical stabilization of the ankle joint while allowing functional sagittal plane motion. The AO was an elastic ankle support (neoprene material) that started from approximately 5 cm above the lateral malleolus to the base of the metatarsal bones with one medial and one lateral stay (Tanyar, Tehran, Iran). The free toes and heel region allowed for a full range of movement (Figure 1a). The brace size was determined based on the ankle circumference. An ankle circumference of 16–20 cm corresponded to size small, 20–24 cm to medium, 24–28 cm to large, and 28–32 cm to extra-large.

The vibration system consisted of four miniature coin-type eccentric rotating mass (ERM) vibration motors (model 310-103, Precision Micro drives Ltd., London, UK) integrated into an elastic ankle band worn circumferentially around the ankle joint. Each motor had a diameter of 10 mm and a thickness of 3.4 mm and operated at a rated voltage of 3 V DC (operating range: 2–3.5 V). These motors were selected due to their stable output characteristics, low noise, and established use in wearable haptic and biomedical research [28,29]. The four vibration motors were positioned over the anterior, posterior, medial, and lateral aspects of the ankle joint to provide distributed cutaneous stimulation. Motors were placed over the tibialis anterior tendon (anterior), the Achilles paratenon (posterior), the deltoid ligament region (medial), and the anterior talofibular and calcaneofibular ligament region (lateral) [29]. This area was marked on the elastic band during initial assessment. The elastic band ensured consistent skin contact while allowing unrestricted ankle movement. Vibration delivery was controlled by a microcontroller-based unit (Arduino Nano, ATmega328P; Arduino, Monza, Italy), operating at 16 MHz with a 5 V logic level. The Arduino IDE software (Version 2.0, Arduino, Ivrea, Italy) was programmed to output a stable vibration frequency, ensuring consistent temporal stimulation throughout the intervention. Although the frequency was tightly regulated by the microcontroller, the displacement amplitude at the skin interface was defined by the manufacturer’s specifications for the actuator component, and the study did not measure real-time in vivo transmission variations. Motor actuation was managed through a dedicated haptic driver integrated circuit (DRV2605L; Texas Instruments, Dallas, TX, USA), which enabled precise and repeatable control of vibration characteristics. Pulse-width modulation (PWM) was used to regulate vibration intensity and timing.

The system was powered by two rechargeable lithium-polymer batteries (3.7 V each, connected in series to provide 7.4 V), with voltage regulated to 5 V using a step-down buck converter (LM2596; Texas Instruments, Dallas, TX, USA). All electronic components, including the microcontroller, motor driver, battery, and voltage regulator, were housed in a compact ABS enclosure measuring approximately 10 cm × 10 cm × 3 cm, which was attached to the elastic band on the lateral aspect of the lower leg (Figure 1b). Vibration stimulation was delivered at a mid-frequency range of 80–100 Hz, which is considered optimal for activating Meissner and Pacinian corpuscles cutaneous mechanoreceptors [29]. Vibration amplitude was maintained within a low-amplitude range of approximately 0.1–0.4 mm to provide effective sensory input while minimizing discomfort, fatigue, or sensory overload [18]. An alternating quadrant vibration pattern with intermittent pauses was employed to enhance spatial sensory input around the ankle joint [29]. In this pattern, the medial and lateral vibration motors were activated simultaneously for 1 s, followed by a 0.5 s pause with no stimulation. Subsequently, the anterior and posterior motors were activated simultaneously for 1 s, followed by another 0.5 s pause. This sequence constituted one complete stimulation cycle and was repeated continuously throughout the intervention period. The timing and sequence of activation were hard-coded into the microcontroller firmware to ensure consistency and reproducibility across participants. To remove the possible impact of different footwear styles on the outcomes, this research employed uniform footwear (Messori Inc., Tehran, Iran) throughout the study process. The shoes featured a low-top athletic profile with a flat insert of uniform density (Shore A ≈ 30).

### 2.5. Outcome Measures

We assessed Vibration Detection Rate (VDR) and phase plane portraits (PPP) as the primary outcome measures. For a secondary outcome, the Static balance performance was evaluated with the Stork Test. Outcome measures were collected at four time points: baseline, after 10 min use of the intervention, two weeks after the intervention, and four weeks after the intervention. All tests were conducted in a randomized sequence to minimize fatigue and learning effects, with one-minute seated rest intervals between trials. The same verbal instructions and environmental conditions were applied for each measurement session to ensure intra-subject and inter-session consistency. All outcome measures were assessed on the affected limb. This unilateral assessment strategy was employed to target the sensorimotor deficits and mechanical instability characteristic of the involved ankle in individuals with unilateral CAI. The assessments were conducted by a single experienced examiner, who is Certified Orthotist with over 5 years of clinical experience in evaluating patients with musculoskeletal disorders. Each evaluation session lasted approximately 40 to 45 min.

#### 2.5.1. Vibration Detection Rate

VDR was assessed using a 128 Hz tuning fork (Surgicon, Sialkot, Pakistan; circular cross-section; dimensions: 120 mm × 17 mm × 5 mm; total length: 15 cm; weight: 30 g). Participants were instructed to remove their shoes and the intervention and then lie comfortably in a supine position on an examination bed. The tuning fork was struck in a consistent manner and applied perpendicularly with light pressure to the skin over four anatomical landmarks encircling the talocrural joint, including the anterolateral region over the anterior talofibular ligament, the posterolateral region posterior to the lateral malleolus near the calcaneofibular ligament, the anteromedial region anterior to the medial malleolus over the deltoid ligament area, and the posteromedial region posterior to the medial malleolus adjacent to the tarsal tunnel. At each site, participants were instructed to verbally report both the onset and cessation of vibration perception. The vibration detection rate, expressed as the percentage of correctly identified vibration stimuli across five repeated trials at each site (20 trials per ankle), was calculated to quantify vibration perception. Previous studies have demonstrated acceptable reliability and validity of the 128 Hz tuning fork for the clinical assessment of vibration perception in the lower extremities, with reported ICC ranging from 0.78 to 0.93, sensitivity of approximately 80–90%, and specificity of 85–95% for detecting sensory impairment [30].

#### 2.5.2. Phase Plane Portraits

Balance was assessed using a Kistler force plate (BA 9286, Kistler, Winterthur, Switzerland). Data were sampled at 50 Hz and low-pass filtered using a Butterworth filter with a cutoff frequency of 6 Hz. Participants were instructed to stand on the force plate with the affected limb while the contralateral limb was lifted off the platform, maintaining approximately 70° of knee flexion and 30° of hip flexion. During testing, participants were instructed to stand in the position, with arms relaxed at their sides, and eyes focused on a fixed target at eye level. A trial was considered failed and discarded if the participant lost balance, stepped off the plate, or touched the ground with the other foot; in such cases, the trial was repeated. Data from three successful trials were averaged to ensure stability of the results. Each trial lasted 25 s, and the initial 5 s was excluded from the analysis to account for postural stabilization. Force plate assessment is considered a valid and reliable method for the quantitative assessment of postural stability, with reported ICC ranging from 0.80 to 0.95 for CoP measures during single-leg stance tasks [31].

The phase plane portrait (PPP) is a CoP-based postural stability metric that characterizes both the static and dynamic components of postural control by simultaneously considering CoP displacement and velocity. Unlike conventional CoP measures that evaluate position or velocity independently, the phase plane approach integrates these variables to provide a more reliable representation of postural control dynamics. Riley et al. (1995) demonstrated that combining displacement and velocity information improves the sensitivity of postural stability assessment. Furthermore, the test–retest reliability of the phase plane portrait in both the anteroposterior (AP) and mediolateral (ML) directions during quiet standing has been reported to be acceptable [32]. The phase plane portrait was computed by plotting CoP displacement against its corresponding velocity. For each direction, the phase plane value was calculated as the resultant magnitude of normalized CoP position and velocity. The phase plane portrait parameters were calculated using the following formulas [33]:

For AP direction:

$${\mathrm{PPP}}_{\mathrm{AP}}\left(i\right)=\sqrt{{\left(\frac{\frac{{-M}_{Y}\left(i\right)}{F_{Z}(i)}-{\bar{X}}_{\mathrm{AP}}}{{\mathrm{SD}}_{\mathrm{AP}}}\right)}^{2}+{\left(\frac{\frac{\left(\frac{{-M}_{Y}\left(i+1\right)}{F_{Z}(i+1)}-{\bar{X}}_{\mathrm{AP}}\right)-\left(\frac{{-M}_{Y}\left(i-1\right)}{F_{Z}(i-1)}-{\bar{X}}_{\mathrm{AP}}\right)}{2∆t}}{{\mathrm{SD}}_{V\;\mathrm{AP}}}\right)}^{2}}$$

For ML direction:

$${\mathrm{PPP}}_{\mathrm{ML}}\left(i\right)=\sqrt{{\left(\frac{\frac{M_{X}\left(i\right)}{F_{Z}(i)}-{\bar{X}}_{\mathrm{ML}}}{{\mathrm{SD}}_{\mathrm{ML}}}\right)}^{2}+{\left(\frac{\frac{\left(\frac{M_{X}\left(i+1\right)}{F_{Z}(i+1)}-{\bar{X}}_{\mathrm{ML}}\right)-\left(\frac{M_{X}\left(i-1\right)}{F_{Z}(i-1)}-{\bar{X}}_{\mathrm{ML}}\right)}{2∆t}}{{SD}_{V\;\mathrm{ML}}}\right)}^{2}}$$

For AP-ML direction:

$${\mathrm{PPP}}_{\mathrm{Total}}\left(i\right)=\sqrt{{\left({\mathrm{PPP}}_{\mathrm{AP}}(i)\right)}^{2}+{\left({\mathrm{PPP}}_{\mathrm{ML}}(i)\right)}^{2}}$$

- M_X_(i) and M_Y_(i) denote the moments about ML and AP axes at sample i, respectively.
- Fz(i) represents the vertical ground reaction force at sample i.

#### 2.5.3. Stork Test

In the Stork Test, participants stood on the affected limb with both hands placed on the iliac crests. The non-affected foot was positioned against the medial aspect of the knee of the stance limb. Participants were then instructed to raise the heel of the stance foot from the ground, maintaining balance on the forefoot. Timing commenced at the moment the heel of the stance foot lifted off the ground and continued until one of the following termination criteria occurred: removal of the hands from the iliac crests, movement or rotation of the stance foot, loss of contact between the non-supporting foot and the knee of the stance limb, or the heel of the stance foot touching the ground again. The total duration (in seconds) for which the participant maintained the position was recorded as the test outcome. The Stork Test has demonstrated excellent intra- and inter-rater reliability for detecting postural control deficits in individuals with CAI (ICC > 0.85) [34].

### 2.6. Statistical Analysis

Sample size estimation was performed using G Power* software (version 3.1.9.2; Heinrich Heine University, Düsseldorf, Germany). The calculation was based on a moderate effect size (Cohen’s d = 0.32) for improvements in the CoP anteroposterior phase plane portrait following AO intervention in individuals with CAI. A total of 30 participants were included in the final analysis according to the intention-to-treat principle. Cohen’s d was derived using the formula where the difference between the mean change scores of the two groups is divided by the pooled standard deviation. In the reference study, the intervention group demonstrated a mean change of 5.84 cm/s (SD = 1.12) and the control group showed a mean change of 5.49 cm/s (SD = 1.08). The resulting pooled standard deviation was approximately 1.10, yielding an effect size of 0.32 (0.35/1.10) [35]. To align with the study design, a repeated measures ANOVA (test family: F-tests) was specified, involving two groups and four measurement time points. The calculation assumed a correlation of 0.5 among repeated measures and a non-sphericity correction of 1. With an alpha level of 0.05 and a power of 0.80, the minimum required sample size was 24 participants. To account for a projected 20% attrition rate, a total of 30 participants were recruited (15 per group) and included in the final analysis according to the intention-to-treat principle. Consistent with established clinical trial methodology, the calculation was based on the primary biomechanical outcome of anteroposterior center of pressure dynamics, utilizing a moderate effect size reported in prior trials involving CAI population. This approach ensures adequate power for the most clinically relevant primary endpoint, while secondary outcomes are interpreted with appropriate consideration of their exploratory nature and observed effect sizes. The power analysis accounted for the longitudinal nature of the data, considering the four time points and an anticipated within-subject correlation. The final sample size (N = 30) provided sufficient power to detect a moderate interaction effect. Furthermore, an intention-to-treat (ITT) analysis was employed for all participants, ensuring that the statistical power was maintained and potential attrition bias was mitigated throughout the 4-week follow-up period.

The analysis strategy was based intension to treat (ITT) strategy. ITT approach was applied, whereby all randomized participants were included in the final analysis. For participants with missing post-intervention data due to loss to follow-up, missing values were imputed using the mean value of the respective outcome within their assigned group. This method ensured that all 30 participants remained in the analysis set. Changes in outcomes over time were analyzed using repeated measures analysis of variance (RM-ANOVA), with group (AO + vibration vs. AO alone) as the between-subjects’ factor and time (baseline, immediately post-intervention, 2-week follow-up, and 4-week follow-up) as the within-subjects’ factor. In this model, the interaction effect of group × time was examined to detect between-group differences at each time point. The assumption of sphericity was examined using Mauchly’s test. In cases where sphericity was violated, the Greenhouse-Geisser correction was applied to adjust the degrees of freedom. A two-tailed significance level of *p* < 0.05 was considered statistically significant for omnibus tests. Given the inclusion of five outcome variables, Bonferroni-corrected pairwise comparisons were conducted following significant omnibus effects to control for type I error inflation. Accordingly, the adjusted level of statistical significance was set at *p* < 0.01 for post hoc analyses. Effect sizes (partial η^2^) and confidence intervals were reported to facilitate clinical interpretability. Effect sizes were interpreted according to thresholds (small ≈ 0.01, moderate ≈ 0.06, large ≥ 0.14) [36].

For the primary RM-ANOVA, a *p*-value of less than 0.05 was considered statistically significant for each independent outcome measure. The Bonferroni adjustment was reserved specifically for the post hoc pairwise comparisons to maintain the family-wise error rate across multiple time points, as each outcome was hypothesized to represent a distinct component of the sensorimotor system.

## 3. Results

### 3.1. Participant Flow and Baseline Data

**Figure 2:**
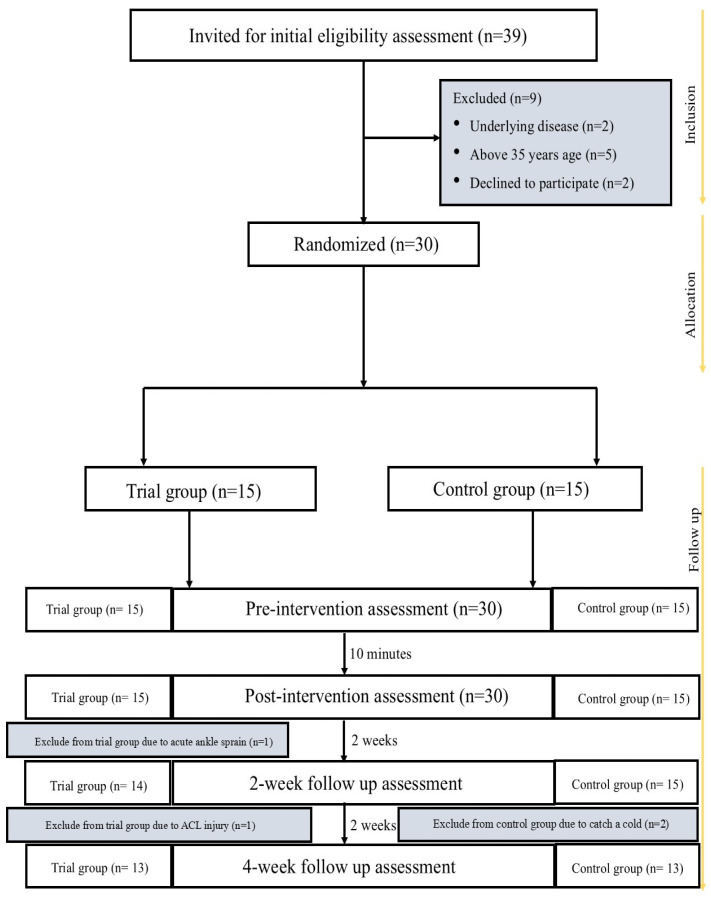
Participant flow diagram.

Participant flow through the study is illustrated in accordance with the CONSORT guidelines (Figure 2). In accordance with ITT principle, all 30 randomized participants were included in the analysis. No serious adverse events, no skin irritation, no discomfort requiring discontinuation, and no vibration-related neurological or sensory complaints were reported in either group. Overall, the intervention was well tolerated, and no safety concerns associated with the use of the vibration band in combination with the AO were identified. Baseline demographic and clinical characteristics are shown in Table 1.

### 3.2. Outcomes and Estimation

A repeated-measures ANOVA revealed outcome-specific intervention effects (Table 2). The ankle orthosis plus vibration band group demonstrated significantly greater improvement over time than the orthosis group in VDR detection rate (F (3,26) = 31.93, *p* < 0.001, η^2^ = 0.78), with an approximate 7% increase from baseline to week 4 compared with nearly 3% in the control group. This represents a large interaction effect, indicating a superiority of the combined intervention for sensory performance.

A significant interaction was also observed for the anteroposterior phase plane portrait (F (3,26) = 3.53, *p* = 0.02, η^2^ = 0.29). The combined intervention was associated with an approximate 43% reduction over time, whereas the orthosis group showed only an 8% reduction, reflecting a moderate-to-large effect in favor of the combined treatment for anteroposterior postural stability.

In contrast, no significant time × group interaction was found for the mediolateral phase plane portrait (F (3,26) = 0.32, *p* = 0.80, η^2^ = 0.03), total phase plane portrait score (F (3,26) = 0.09, *p* = 0.96, η^2^ = 0.01), or Stork test time (F (3,26) = 0.53, *p* = 0.66, η^2^ = 0.06). The associated small effect sizes indicate that the observed temporal variations in these measures were not meaningfully different between groups.

For VDR detection rate, no significant between-group differences were observed at pre-intervention (*p* = 0.63), post-intervention (*p* = 0.11), or 2-week follow-up (*p* = 0.17). However, by 4 weeks, the comparison reached statistical significance (MD = 3.13 ± 0.62, 95% CI:1.84 to 4.41, *p* < 0.001), with a large effect size (d = 1.82). This pattern indicates that the superiority of vibro-stimulation did not emerge immediately, but became pronounced over time, ultimately yielding a substantial advantage in sensory detection performance. The narrow confidence interval and large effect suggest that this difference was statistically robust.

**Figure 3:**
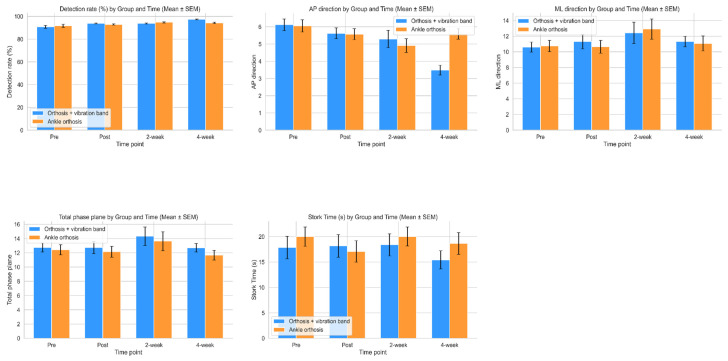
Mean (±SEM) changes in vibration perception detection rate, phase plane parameters (anteroposterior, mediolateral, and total), and stork time across the four assessment points (pre-intervention, post intervention, 2 week follow up, and 4 week follow up) in the orthosis plus vibration band and ankle orthosis groups.

A similar pattern was observed for the anteroposterior phase plane portrait. Between-group differences were non-significant at baseline (*p* = 0.90), post-intervention (*p* = 0.91), and 2-week follow-up (*p* = 0.56), with corresponding effect sizes remaining trivial to small. In contrast, the 4-week follow-up showed a significant difference (MD = −2.10 ± 0.42, 95% CI: −2.96 to −1.23, *p* < 0.001), accompanied by a very large effect size (d = −1.79) (Table 3). The negative direction of the mean difference, together with the magnitude of the effect, indicates a marked between-group separation in favor of the combined intervention at the later stage of follow-up. From a clinical perspective, this finding supports the interpretation that meaningful gains in anteroposterior postural control may require time to consolidate before becoming detectable. Figure 3 illustrates the mean values (±SEM) of the outcome variables across time for both groups.

## 4. Discussion

The present study adds to the emerging evidence advocating integrative interventions that combine orthotic support with vibro-stimulation to enhance sensory-motor function in individuals with CAI. While several recent approaches have primarily focused on passive external ankle supports [16], our findings contribute to the evolving understanding of how coupling mechanical and sensory cues may improve proprioceptive input and postural control in this population. Although previous studies have examined the effects of vibration-based orthotic interventions in populations with musculoskeletal disorders such as osteoarthritis [37], neurological conditions including stroke [38], cerebral palsy [39], and Parkinson’s disease [40], as well as in physically active healthy individuals [41], the clinical value of such an approach in CAI had remained unclear. The present findings help address this gap by showing that, over a 4-week period, the combination of ankle bracing and vibro-stimulation produced significantly greater improvements than ankle bracing alone in VDR detection rate and anteroposterior phase plane portrait. In contrast, no significant between-group differences were observed for mediolateral phase plane portrait, total phase plane portrait score, or Stork test time. These findings suggest that the addition of localized vibration may selectively enhance sensory acuity and some dimensions of postural control, rather than uniformly improving all balance-related outcomes. Beyond statistical significance, it is essential to consider the clinical relevance of our findings. The large effect sizes observed in this study, including the η^2^ = 0.78 for VDR and Cohen’s *d* = 1.79 for the anteroposterior phase plane portrait, underscore the significant impact of the vibro-stimulation ankle brace. These effect sizes suggest that the intervention induces a meaningful improvement in sensory-motor dynamics. Given that patients with CAI frequently suffer from sensory deficits and postural control impairments, these results indicate that the combined vibro-stimulation and bracing approach offers a statistically significant strategy for enhancing functional ankle stability in this population.

The improvement in VDR detection rate in the vibro-stimulation ankle bracing group may be explained by enhanced stimulation of periarticular and musculotendinous receptors around the ankle joint, particularly muscle spindles, Golgi tendon organs, Ruffini joint mechanoreceptors cutaneous receptors, and rapidly adapting mechanoreceptors sensitive to oscillatory input [18]. From a neurophysiological perspective, externally applied vibration may amplify afferent inflow and facilitate transmission within sensory pathways, thereby improving the detection and processing of weak proprioceptive stimuli [42]. The human sensorimotor system behaves as a nonlinear dynamic system, in which subthreshold or low-amplitude sensory input can be functionally enhanced by an appropriately tuned noisy or oscillatory stimulus [29]. Perceptible local vibration may increase synchronous neural firing and membrane depolarization in sensory fibers, thereby improving somatosensory discrimination [18].

The finding that the combined intervention also improved anteroposterior phase plane portrait to a greater extent than orthosis alone is biomechanically meaningful. CAI is associated with altered postural strategies, reduced sensorimotor efficiency, and impaired regulation of COP [9,10]. A neoprene AO may mechanically enhance talocrural and subtalar stability through external compression and mediolateral support, thereby reducing excessive joint excursion and the need for rapid corrective postural adjustments [10]. When this mechanical support is combined with vibration, the intervention may additionally optimize afferent feedback from the ankle–foot complex, improving the timing and coordination of neuromuscular responses [21]. Anteroposterior sway in quiet or semi-static stance is more strongly modulated by ankle strategy than mediolateral sway [43]. Because the ankle joint plays a dominant role in controlling forward–backward body oscillations, interventions targeting ankle biomechanics and proprioceptive input would be expected to exert a more direct effect on anteroposterior postural regulation [41]. In the present study, this interpretation is supported by the significantly greater reduction in AP phase plane portrait in the combined group, particularly by week 4, with a very large between-group effect size. Some studies suggests that this population may exhibit altered corticospinal excitability and higher resting motor threshold in muscles such as the peroneus longus [44]. Localized vibration, particularly when applied near the peroneal musculotendinous structures, may activate muscle spindle Ia afferents and facilitate reflexive or supraspinally mediated muscle activation [21]. This facilitation may reduce sensorimotor delay and improve the responsiveness of the ankle stabilizing musculature during postural tasks. Moreover, vibration-induced enhancement of spatiotemporal sensory input may improve the central integration of afferent signals and contribute to more postural corrections [20]. Some evidences indicating that vibro-stimulation insoles can decrease jump performance time in young, healthy, and physically active populations [41].

Mediolateral postural control is less dependent on the ankle strategy alone and may rely more on hip strategy, trunk control, and whole-body coordination [43]. Therefore, an intervention primarily targeting ankle stability and local sensory input may have limited influence on the neuromechanical processes governing mediolateral balance. Also, in CAI, compensatory movement patterns such as increased in ground contact time, increased dorsiflexion of the first metatarsophalangeal joint, increased foot supination, and lateralization of pressure of the lateral edge of the mid foot and the forefoot may be more chronic and centrally integrated, making them less responsive to a relatively short-duration peripheral intervention [15]. In other words, the absence of significant findings in mediolateral and total indices may also reflect limited statistical power for smaller effects, especially given the very small interaction effect sizes reported for these variables.

Despite previous reports that AOs can produce immediate improvements in clinical balance tests such as the Star Excursion Balance Test or single-limb stance tasks [17], the present study did not detect a significant effect on Stork test time. One likely explanation is that Stork performance reflects the integrated contribution of multiple segments and systems, including the ankle, knee, hip, trunk, and central postural control mechanisms [34]. As a result, interventions that primarily modify ankle biomechanics and local sensory feedback over a short period may not be sufficient to induce a measurable change in this more complex functional test. In addition, the Stork test may have lower sensitivity to subtle sensorimotor improvements than instrumented postural metrics such as phase plane analysis. It is also possible that the vibration parameters used in this study were insufficient to generate a clinically detectable enhancement in functional balance performance [18].

For both VDR detection rate and anteroposterior phase plane portrait, between-group differences were not significant immediately post-intervention or at the 2-week follow-up, but became significant at 4 weeks. This delay suggests that the effects of combined orthosis–vibration rehabilitation may be after adaptive rather than immediate [19]. Such a pattern may reflect gradual sensory recalibration, repeated exposure-driven adaptation of afferent processing, or progressive optimization of postural control strategies over time [12,31].

This study has several limitations. First, the sample consisted of young, physically active individuals; therefore, the generalizability of the findings to older adults, less active populations, or individuals with comorbidities remains uncertain. Second, the neuromuscular mechanisms underlying the observed effects were not directly assessed using objective tools such as electromyography, reflex testing, or measures of corticospinal excitability. Third, the study focused on immediate and short-term outcomes, and the medium- and long-term persistence of the intervention effects is still unknown. Future research should investigate different vibration patterns and orthotic designs, and whether combining this approach with active exercise-based rehabilitation could yield superior improvements. Fourth, although the sample size was determined a priori for the primary outcome using a single effect size input, as is standard practice in clinical trial design, the study may have been insufficiently powered to detect small intervention effects for secondary biomechanical and functional measures. The negligible effect sizes observed for mediolateral phase plane portrait, total phase plane portrait score, and Stork test performance, however, support the interpretation that the combined intervention did not meaningfully influence these specific outcomes within the study timeframe. Future multicenter trials with larger cohorts should prospectively power secondary endpoints to confirm these null findings. Fifth, a key limitation of this study is the inability to blind participants to the intervention, given the distinct sensory nature of the vibro-stimulation system. This lack of blinding could potentially influence subjective outcomes, such as the vibration perception threshold, through expectation effects. Although we employed a standardized testing protocol and refrained from discussing expected outcomes with participants to minimize this risk, the possibility of performance bias cannot be entirely ruled out. Sixth, the use of a 128 Hz tuning fork for evaluating vibration perception is supported by previous literature as a valid and reliable clinical assessment tool. While it remains standard practice for its clinical utility and accessibility, we acknowledge that it is inherently more subjective than automated quantitative sensory testing. Although we standardized the application and testing protocol to minimize variability, we recognize that the reliance on patient reporting introduces a degree of subjectivity. Furthermore, the absence of a sham vibration condition means that we cannot definitively rule out the role of a placebo effect. While our results show that the addition of vibro-stimulation to semi-rigid bracing is associated with improved tactile sensation and anteroposterior postural control in individuals with CAI, the design does not allow for a pure isolation of the vibro-stimulation effect. Future studies utilizing a double-blind, sham-controlled design are warranted to isolate the precise physiological contribution of vibro-stimulation to sensorimotor recovery.

## 5. Conclusions

This randomized clinical trial indicates that adding vibro-stimulation to AO use may provide additional benefit over AO alone in individuals with CAI, particularly for VDR and anteroposterior phase plane portrait. However, these benefits did not extend to ML stability, total phase plane portrait score, or Stork performance. This study shows that combined orthotic-sensory rehabilitation may target sensorimotor deficits in CAI, while also highlighting the need for multimodal or longer-duration strategies to influence more complex and different aspects of postural function.

## Figures and Tables

**Table 1:** The characteristics of participants.

Characteristics	Ankle Orthosis + Vibration Band M ± SD ^1^	Ankle Orthosis M ± SD	*p* Value
Body mass (kg)	65.3 ± 6.8	66.2 ± 10.6	0.81
Height (cm)	176.1 ± 4.9	175 ± 3.2	0.43
Age (years)	20.8 ± 1.2	20.1 ± 1.7	0.19
Body mass index (kg/m^2^)	22.0 ± 2.2	23.1 ± 3.8	0.29
Sex (male/female)	7/8	5/10	0.29
CAIT score (0–30)	18.3 ± 3.1	20.1 ± 1.9	0.16
Time since last ankle sprain (months)	3.2 ± 1.9	3.9 ± 2.2	0.35
Affected side (right/left)	13/2	12/3	0.69
Dominant side (right/left)	14/1	13/2	0.55
Number of previous ankle sprains	6.2 ± 3.02	5.52 ± 2.83	0.58
FAAM-ADL (%)	87.02 ± 10.27	85.67 ± 11.36	0.75
FAAM-Sport (%)	67.32 ± 15.01	65.42 ± 19.22	0.74

^1^ M: Mean, SD: Standard Deviation.

**Table 2:** Descriptive statistics, encompassing means and standard deviations for each variable stratified by group at various study time points, alongside the inferential results derived from the RM-ANOVA, are presented.

Outcome	Groups	Assessments				Repeated Measures ANOVA
		Pre M ± SD	Post M ± SD	2-Week M ± SD	4-Week M ± SD	Time × Group Effects
Vibration Detection Rate						
Detection rate (%)	Vibro-stimulation ankle bracing (n = 15)	90.8 ± 5.04	93.86 ± 1.55	93.73 ± 1.83	97.4 ± 1.59	Wilks’ Lambda = 0.21 F _(3,26)_ = 31.93 * *p* ˂ 0.001 η^2^ = 0.78
	Ankle bracing (n = 15)	91.66 ± 4.90	92.93 ± 1.62	94.73 ± 2.12	94.26 ± 1.83	
Phase plane portraits						
Anteroposterior direction	Vibro-stimulation ankle bracing (n = 15)	6.12 ± 1.33	5.62 ± 1.22	5.29 ± 1.96	3.49 ± 1.10	Wilks’ Lambda = 0.7 F _(3,26)_ = 3.53 * *p* = 0.02 η^2^ = 0.29
	Ankle bracing (n = 15)	6.05 ± 1.38	5.57 ± 1.24	4.91 ± 1.55	5.59 ± 1.20	
Mediolateral direction	Vibro-stimulation ankle bracing (n = 15)	10.61 ± 2.42	11.30 ± 3.60	12.42 ± 5.32	11.31 ± 2.53	Wilks’ Lambda = 0.96 F _(3,26)_ = 0.32 *p* = 0.8 η^2^ = 0.03
	Ankle bracing (n = 15)	10.75 ± 2.81	10.66 ± 3.17	12.91 ± 5.04	11.07 ± 2.75	
Total	Vibro-stimulation ankle bracing (n = 15)	12.75 ± 2.44	12.73 ± 3.30	14.33 ± 5.05	12.71 ± 2.31	Wilks’ Lambda = 0.98 F _(3,26)_ = 0.09 *p* = 0.96 η^2^ = 0.01
	Ankle bracing (n = 15)	12.43 ± 2.77	12.15 ± 2.97	13.63 ± 5.15	11.66 ± 2.60	
Stork						
Time (s)	Vibro-stimulation ankle bracing (n = 15)	17.86 ± 8.61	18.19 ± 8.59	18.40 ± 8.40	15.43 ± 6.85	Wilks’ Lambda = 0.93 F _(3,26)_ = 0.53 *p* = 0.66 η^2^ = 0.06
	Ankle bracing (n = 15)	20.02 ± 7.34	17.07 ± 8.17	20.02 ± 7.24	18.66 ± 8.34	

* *p* was considered significant at ˂0.05.

**Table 3:** The results of pairwise comparisons across different study time intervals for each outcome, along with the findings from RM-ANOVA (comparing results for trial vs. control), are presented.

Outcome	Time	MD ± SE (95% CI)	*p*	Effect Size (Cohens’ d)
Vibration Detection Rate				
Detection rate (%)	Pre	−0.86 ± 1.81 (−4.59 to 2.85)	0.63	−0.17
	Post	0.93 ± 0.58 (−0.25 to 2.12)	0.11	0.59
	2-week Follow up	−1.00 ± 0.72 (−2.48 to 0.48)	0.17	−0.5
	4-week Follow up	3.13 ± 0.62 (1.84 to 4.41)	* ˂0.001	1.82
Phase plane portraits				
Anteroposterior	Pre	0.06 ± 0.49 (−0.95 to 1.08)	0.9	0.05
	Post	0.05 ± 0.45 (−0.87 to 0.97)	0.91	0.04
	2-week Follow up	0.37 ± 0.64 (−0.94 to 1.70)	0.56	0.22
	4-week Follow up	−2.1 ± 0.42 (−2.96 to −1.23)	* ˂0.001	−1.79

* *p* was considered significant at ˂0.01.

## Data Availability

The data supporting the findings of this study are available from the corresponding authors upon reasonable request. Due to privacy and ethical restrictions, the data are not publicly deposited.
